# The relationship between breast cancer molecular subtypes and mast cell populations in tumor microenvironment

**DOI:** 10.1007/s00428-017-2103-5

**Published:** 2017-03-18

**Authors:** Anna Glajcar, Joanna Szpor, Agnieszka Pacek, Katarzyna Ewa Tyrak, Florence Chan, Joanna Streb, Diana Hodorowicz-Zaniewska, Krzysztof Okoń

**Affiliations:** 1 Department of Pathomorphology, Jagiellonian University Medical College, ul. Grzegórzecka 16, 31-531 Kraków, Poland; 2 2nd Department of Internal Medicine, Jagiellonian University Medical College, Kraków, Poland; 3 Department of Oncology, Jagiellonian University Medical College, Kraków, Poland; 4 Department of General, Oncological, and Gastrointestinal Surgery, Jagiellonian University Medical College, Kraków, Poland

**Keywords:** Mast cells, Breast cancer, Molecular classification

## Abstract

Mast cells (MCs) are a part of the innate immune system. The MC functions toward cancer are partially based on the release of chymase and tryptase. However, the MC effect on breast cancer is controversial. The aim of our study was to investigate the presence of MCs in breast cancer tumors of different molecular subtypes and their relationships with other pathological prognostic factors. Tryptase- and chymase-positive mast cell densities were evaluated by immunohistochemistry in 108 primary invasive breast cancer tissue samples. Positive cells were counted within the tumor bed and at the invasive margin. For all analyzed MC subpopulations, we observed statistically significant differences between individual molecular subtypes of breast cancer. The significantly higher numbers of intratumoral chymase- and tryptase-positive mast cells were observed in luminal A and luminal B tumors compared to triple-negative and HER2+ non-luminal lesions. A denser MC infiltration was associated with lower tumor grade, higher ER and PR expression, lower proliferation rate as well as the lack of HER2 overexpression. The results obtained in our study indicate a possible association of chymase- and tryptase-positive MCs with more favorable cancer immunophenotype and with beneficial prognostic indicators in breast cancer.

## Introduction

Breast cancer is the most common cancer in females in the developed world. It is heterogeneous in terms of prognosis, morphology, and molecular biology; on the basis of its gene expression pattern, four main molecular subtypes were distinguished: luminal A, luminal B, HER2 non-luminal, and basal-like. This classification may be emulated by an immunohistochemical panel, which became a standard in routine pathology [1, 2].

Tumor microenvironment consists of fibroblast, endothelial, and immune cells as well as extracellular matrix (ECM) in the immediate surroundings of cancer. It influences anti-tumor host defense, tumor development, neoangiogenesis, and metastatic propensity, and may affect patient’s outcome [1–3].

Mast cells (MCs) are bone marrow-derived cells commonly associated with allergic reactions and responses to parasitic infestations. MC granules store numerous mediators, including heparin, histamine, proteases, chemokines, and growth factors, which are released upon MC activation and contribute to tissue repair, wound healing, and angiogenesis. They modulate functions of other immune cells by either enhancing immunologic response or inducing immune tolerance. MCs are also one of the first cells to infiltrate cancer and can either promote or suppress tumor growth [4–8].

Proteases constitute approximately one fourth of MCs protein content. Based on the expression of chymase and tryptase, the mast-cell-specific serine proteases, human MCs are divided into MC_T_, which expresses only tryptase and MC_TC_, which expresses both tryptase and chymase. These populations predominate in different anatomical locations and vary according to their functions [4, 6, 9]. Tryptase participates in ECM remodeling and is a potent proangiogenic factor, in part by protease-activated-receptor 2 (PAR-2) activation [9–11]. MC tryptase was also reported to activate tumor-associated fibroblasts [12]. Chymase is thought to be important mainly for ECM remodeling; however, it may also induce angiogenesis by activating metalloproteinases (MMPs), such as MMP-9, which releases proangiogenic mediators from stroma [6, 12, 13].

Some MC proteases are stored in complexes with heparin [14]. Heparin suppresses proliferation and reduces the number of breast cancer cell colonies. It was hypothesized that heparin might interrupt interactions between tumor-associated fibroblasts and cancer cells, thus impairing tumor development [15].

The aim of the study was to investigate the density of MCs expressing tryptase and chymase in breast cancers of different molecular subtypes and to examine their relationships with more standard prognostic factors.

Preliminary results from this study were presented at the 6th Jagiellonian University Medical College Doctoral Students’ Conference.

## Materials and methods

### Materials

The material consisted of routinely processed, formalin-fixed paraffin-embedded primary invasive breast carcinomas diagnosed between 2002 and 2014. The archival hematoxylin–eosin-stained slides were re-evaluated and representative, well-preserved specimens were chosen for immunohistochemistry. For nuclear grading, Nottingham Histologic Grade system was used, while staging was performed according to 2010 AJCC system [16].

### Immunohistochemistry

Immunohistochemistry for tryptase, chymase, estrogen receptor (ER), progesterone receptor (PR), and Ki67 protein was performed according to the protocol routinely used in our laboratory. The selected blocks were cut into 4-μm-thick sections. Antigen retrieval was performed by incubating the slides in citrate buffer (pH 6.0; 0.01 M) or EDTA (pH 8.0; 0.01 M) at 97 °C in a water bath for 40 and 30 min, respectively, or by enzymatic digestion with proteinase (21 °C, 7 min). Primary antibodies used in the study are listed in Table 1.

**Table 1:** Antibodies used in the study

	Clone	Dilution	Antigen retrieval	Incubation time (min)	Manufacturer
Tryptase	AA1	1:100	Proteinase	60	Novocastra (Leica Biosystems, Germany)
Chymase	CC1	1:100	Citrate	30	LabVision (ThermoScientific, USA)
Estrogen receptor	6F11	1:25	Citrate	60	Novocastra (Leica Biosystems, Germany)
Progesterone receptor	PgR636	1:50	Citrate	60	Dako, USA
Ki67	MIB-1	1:100	EDTA	60	Dako, USA
HER2/neu	PATHWAY4B5				Ventana Medical System Inc., USA

UltraVision Quanto detection system (LabVision; ThermoScientific, USA) and 3,3′-diaminobenzidine as chromogen were used, and the slides were counterstained with Mayer hematoxylin (Thermo Fisher Scientific, Waltham, USA) and coverslipped.

Immunohistochemistry for HER2 was performed on BenchMark BMK Classic autostainer (Ventana, USA) using UltraVIEW DAB Detection Kit (Ventana Medical Systems Inc., USA).

For specimens with HER2 status 2+ by immunohistochemistry, fluorescence in situ hybridization (FISH) was conducted. FISH was performed using a PathVysion HER-2 DNA Probe Kit II (Abbott Molecular, USA) according to the manufacturer’s protocol. In short, paraffin blocks were cut into 4-μm-thick sections. Hybridization was performed at 37 °C for 14 to 18 h with a locus specific identifier (LSI) DNA probe (~226 kb) SpectrumOrange directly labeled (Abbott Molecular, USA) and a Chromosome Enumeration Probe 17 (CEP17) satellite DNA probe (~5.4 kb) SpectrumGreen directly labeled (Abbott Molecular, USA). 4,6-Diamino-2-phenylidole was used as nuclear counterstain. The LSI HER-2/neu and CEP17 signals were counted on fluorescence microscope equipped with specific filter sets and HER-2/neu to CEP17 ratio >2.0 was considered as HER2/neu overexpression [17].

### Evaluation of immunostaining

The slides stained for tryptase and chymase were scanned on Nikon Labophot-2 optical microscope (Tokyo, Japan) at low magnification (×100), and the areas with the highest number of positive cells were chosen. Then, positively stained cells were counted in five high-power fields (HPF) (400 × 0.2 mm^2^ field area), which represented 1 mm^2^ of the examined tissue. The positive cells located no further than 1 HPF from the tumor edge were regarded as invasive margin, while positive cells located within neoplastic tissue further than 1 HPF from the tumor edge inwards were considered as intratumoral population.

Positive ER and PR expression were set when ≥1% of neoplastic cells showed positive immunostaining. The threshold for discriminating between low and high Ki67 expression was set at ≥14% of positive cells. Scoring of the HER2 stain was performed by standard method [17].

### Definition of breast cancer molecular subtypes

The cases were classified into molecular subtypes according to St Gallen 2013 International Expert Consensus: luminal A (ER+ and PR ≥20%, Ki67 < 14%, HER2−), luminal B/ HER2− (ER+, HER2− with PR <20% and/or Ki67 ≥ 14%), luminal B/HER2+ (ER+ or PR+, HER2+), HER2+ non-luminal (ER−/PR−/HER2+), and triple-negative breast cancer (ER−/PR−/HER2−) [18].

### Statistical analysis

To assess the differences in positive cells’ infiltrate between groups, ANOVA Kruskal–Wallis and Mann–Whitney *U* tests were performed. The correlations between groups were evaluated by using Spearman rank test. All analyses were performed using Statistica 10 (StatSoft Inc., USA). *p* values <0.05 were considered statistically significant.

## Results

### Study group

The study group consisted of 108 cases. The mean age of patients at the time of diagnosis was 55.3 years, ranging from 29 to 87 years. Sixty cases (55.5%) were stage pT1, 45 cases (41.7%) pT2, and 3 cases (2.8%) pT3. Lymph node status was pN0 in 54 cases (50.0%), pN1 in 31 cases (28.7%), pN2 in 9 cases (8.3%), and pN3 in 13 cases (12.0%).

Distribution of molecular subtypes was as follows: luminal A in 30 cases (27.8%), luminal B/HER2− in 19 cases (17.6%), luminal B/HER2+ in 10 cases (9.3%), HER2+ non-luminal (HER2+) in 20 cases (18.5%), and triple-negative breast cancer (TNBC) in 29 cases (26.8%). On the basis of the histologic type, 91 cases (84.3%) were classified as “not otherwise specified” (NOS), 15 cases (13.9%) as lobular, and 2 cases (1.8%) as “other.” Nottingham Histologic Grade was G1 in 17 cases (15.7%), G2 in 37 cases (34.3%), and G3 in 54 cases (50%). The patients and tumor characteristics are shown in Table 2.

**Table 2:** Clinicopathologic features of the study group

Characteristic	Number of cases	Percent
Age		
Range: 29–87		
Mean: 55.3		
Tumor size		
pT1	60	55.5
pT2	45	41.7
pT3	3	2.8
Lymph node status		
pN0	54	50.0
pN1	31	28.7
pN2	9	8.3
pN3	13	12.0
Nottingham Histologic Grade		
G1	17	15.7
G2	37	34.3
G3	54	50.0
Histological type		
Ductal	91	84.3
Lobular	15	13.9
Other	2	1.8
Molecular subtype		
Luminal A	30	27.8
Luminal B	19	17.6
Luminal B/ HER2+	10	9.3
HER2+ non-luminal	20	18.5
Triple negative	29	26.8

### MC subpopulations in different breast cancer subtypes

**Fig. 1:**
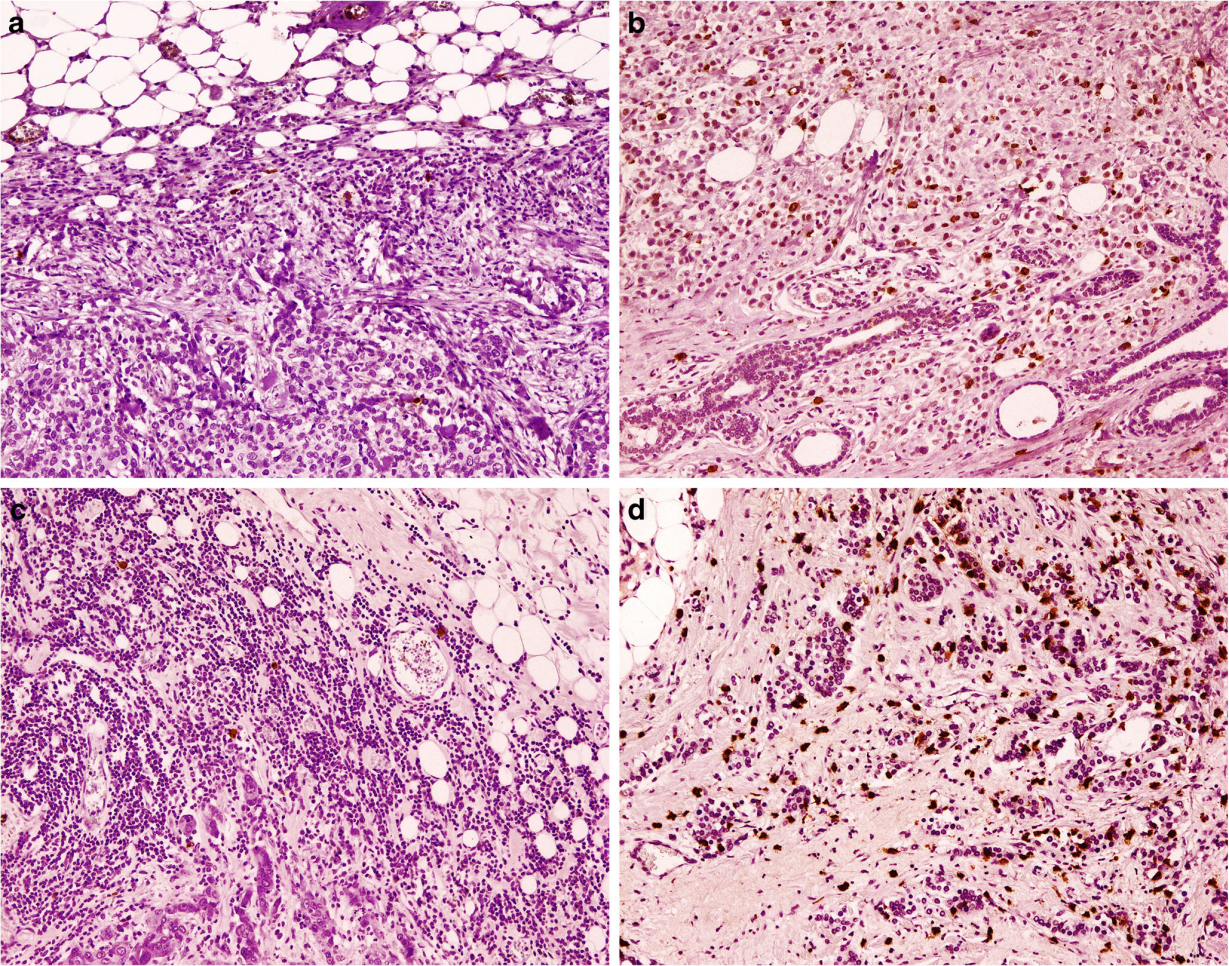
Mast cells in invasive breast cancer. Low (**a**) and high (**b**) chymase-positive mast cells infiltration, low (**c**) and high (**d**) tryptase-positive mast cells infiltration. Immunohistochemistry for tryptase and chymase, magnification ×100

First, we investigated whether the mast cell counts differed between cancers of luminal (ER+ or PR+) and non-luminal (ER− and PR−) immunophenotype. A statistically significant difference was observed for both chymase- and tryptase-positive MCs in either intratumoral location or at the invasive margin (Fig. 1). In all cases, the luminal subtype of tumors was associated with relatively higher MC count (Table 3).

**Table 3:** MC densities in breast cancers of different molecular subtype, immunophenotype, Ki67, and HER2 expression

	Chymase				Tryptase			
	Intratumoral		Invasive margin		Intratumoral		Invasive margin	
	Mean (SD)	*p*	Mean (SD)	*p*	Mean (SD)	*p*	Mean (SD)	*p*
Molecular subtype								
Luminal A	22.59 (10.38)	<0.001	23.21 (7.87)	<0.025	40.40 (16.97)	<0.001	36.27 (20.62)	<0.015
Luminal B	27.72 (12.76)		25.68 (11.95)		36.94 (17.56)		38.74 (19.97)	
Luminal B/HER2+	20.80 (12.45)		19.50 (7.55)		31.10 (20.89)		26.70 (11.49)	
HER2+ non-luminal	15.30 (7.89)		18.70 (9.76)		25.90 (11.43)		24.65 (9.24)	
Triple negative	14.56 (9.82)		19.03 (7.35)		22.50 (13.42)		27.17 (11.28)	
Immunophenotype								
Luminal	23.89 (11.64)	<0.001	23.38 (9.43)	<0.005	37.72 (17.86)	<0.001	35.44 (19.34)	<0.004
Non-luminal	14.87 (8.97)		18.90 (8.32)		23.92 (12.61)		26.14 (10.46)	
HER2 overexpression								
No	20.91 (11.88)	NS	22.25 (9.15)	<0.025	32.99 (17.69)	NS	33.49 (18.01)	<0.015
Yes	17.13 (9.79)		18.97 (8.96)		27.63 (15.08)		25.33 (9.89)	
Ki67 expression								
Low	22.41 (10.97)	NS	22.79 (8.27)	NS	39.17 (16.29)	<0.001	34.89 (19.61)	NS
High	18.56 (11.47)		20.43 (9.44)		27.68 (16.28)		29.22 (14.62)	

**Fig. 2:**
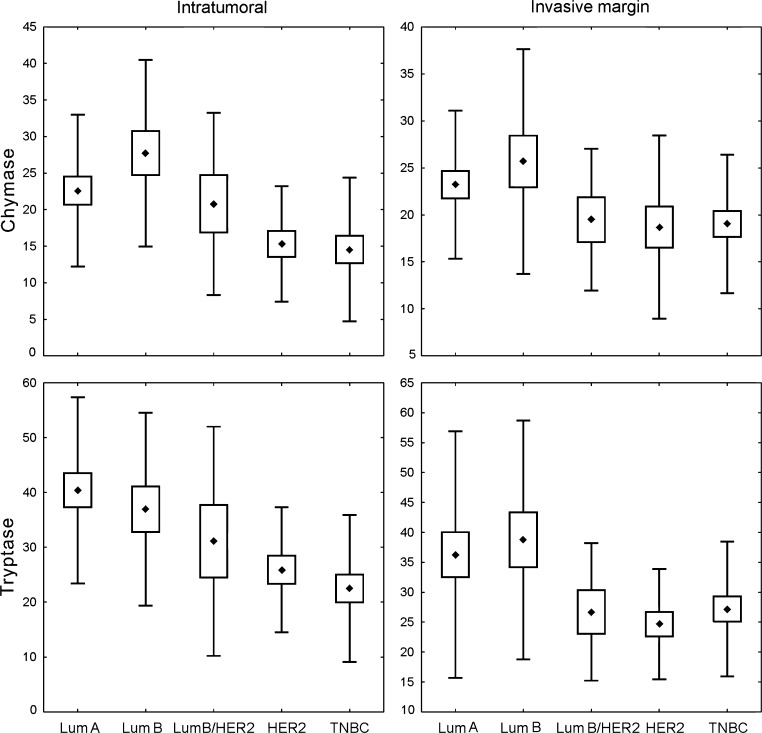
Density of investigated MC subpopulations in breast cancer specimens representing different molecular subtypes: *Lum A* luminal A, *Lum B* luminal B/HER2−, *Lum B/HER2* luminal B/HER2+, *HER2* HER2+ non-luminal, *TNBC* triple-negative subtype. *Central point* is the arithmetic mean, *box* is the arithmetic mean ± standard error, and *whisker* is the arithmetic mean ± standard deviation. ANOVA Kruskal–Wallis test, *p* values are shown in Table 3

Thorough analysis of each of the investigated MC populations showed significant differences in the density of infiltration between molecular subtypes of cancer; this was most evident for intratumoral cells. The number of intratumoral chymase-positive MCs was the highest in luminal B cancers, which differed significantly from TNBC (*p* < 0.002) and HER2+ non-luminal (*p* < 0.025) tumors. Luminal A cancers contained significantly more chymase-positive MCs than TNBC cancers (*p* < 0.04). The intratumoral tryptase-positive MC density was the highest in luminal A tumors and was significantly higher than that in TNBC (*p* < 0.001) and HER2+ non-luminal (*p* < 0.04) cases. The abundance of these cells was also significantly higher in luminal B as compared to TNBC tumors (*p* < 0.015). There was a significant difference in MC density at the invasion front between all the molecular breast cancer subtypes, but no significant difference in post hoc analysis was observed (Fig. 2, Table 3).

The number of MCs at the invasive margin, either chymase- or tryptase-positive, was significantly increased in tumors without HER2 overexpression (*p* < 0.025 and *p* < 0.015, respectively) compared to that in HER2 overexpressed tissues (Table 3).

We also observed also that intratumoral tryptase-positive MCs were strongly associated with tumors of low Ki67 expression (*p* < 0.001) (Table 3).

The numbers of all investigated MC populations showed significant positive correlations with ER and PR expression, as well as a negative correlation with mitotic index. For investigated subpopulations, either in intratumoral area or at the invasion edge, tryptase-positive MCs correlated negatively with Ki67 expression. However, for chymase-positive MCs, such correlation was observed only within the tumor bed.

### MC subpopulations and other pathological prognostic factors

Investigated tumors were stratified according to their size into tumors of diameter ≤2 cm (pT1) and >2 cm (pT > 1). We observed statistically significant differences in tryptase-positive cell densities in both intratumoral compartment (*p* < 0.008) and at the invasion front (*p* < 0.02) between the two groups. The intratumoral tryptase-positive MC density was higher in pT1 tumors (mean 35.5, SD 18.2) as compared to pT > 1 lesions (mean 26.6, SD 14.3). Similarly, for tryptase-positive MCs at the tumor margin, higher density was observed in smaller-sized cancers (pT1—mean 33.7, SD 17.2; pT > 1—mean 28.1, SD 15.2). There were no statistically significant differences in MC densities between cases with and without nodal involvement.

**Fig. 3:**
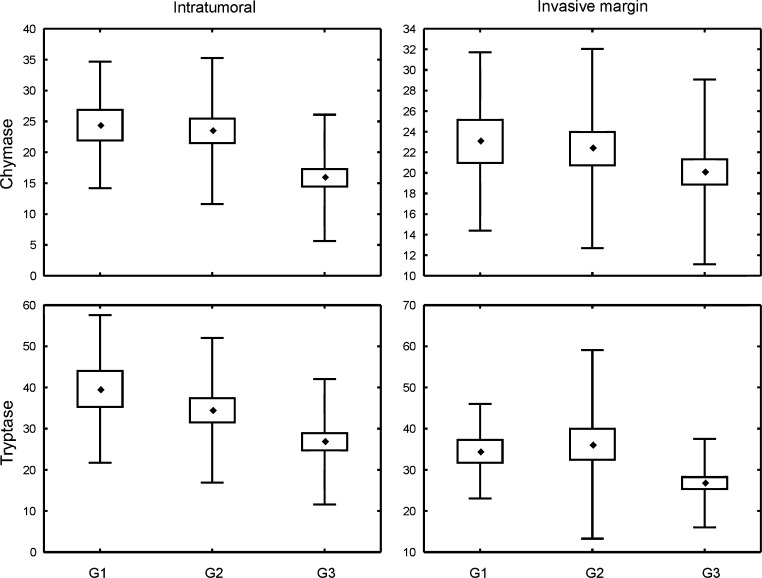
Density of investigated MC subpopulations in breast cancer specimens representing different Nottingham Histologic Grade. *Central point* is the arithmetic mean, *box* is the arithmetic mean ± standard error, and *whisker* is the arithmetic mean ± standard deviation. ANOVA Kruskal–Wallis test, *p* values are shown in Table 4

There were significant differences in the densities of tryptase-positive cells, both in intratumoral compartment and at the invasive margin, as well as intratumoral chymase-positive cell count between tumors of different Nottingham Histologic Grades. The number of intratumoral chymase-positive cells was significantly higher in G1 (*p* < 0.015) and G2 (*p* < 0.008) tumors as compared to G3 lesions. Tryptase-positive MC densities for both intratumoral compartment and invasion front were significantly higher in G1 than in G3 cancers (*p* < 0.015 and *p* < 0.05, respectively) (Fig. 3, Table 4).

**Table 4:** MC density according to tumor grade and breast cancer histological type

	Chymase				Tryptase			
	Intratumoral		Invasive margin		Intratumoral		Invasive margin	
	Mean (SD)	*p*	Mean (SD)	*p*	Mean (SD)	*p*	Mean (SD)	*p*
Nottingham Histologic Grade								
1	24.41 (10.24)	<0.002	23.06 (8.67)	NS	39.65 (17.93)	<0.007	34.53 (11.46)	<0.02
2	23.46 (11.81)		22.36 (9.68)		34.44 (17.59)		36.19 (22.92)	
3	15.87 (10.22)		20.09 (8.98)		26.83 (15.26)		26.78 (10.72)	
Histological type								
NOS	19.07 (11.12)	NS	20.69 (8.57)	NS	30.56 (17.14)	<0.05	29.48 (11.97)	NS
Lobular	24.79 (11.65)		25.71 (12.28)		38.73 (15.93)		42.93 (31.32)	

In respect of tumor histological type, intratumoral tryptase-positive cells were significantly associated with lobular phenotype (Table 4).

## Discussion

The studies concerning MC infiltration in various breast cancer molecular subtypes were scarce and the results were encumbered by varied subtype classifications. In our study, we noted that chymase- and tryptase-positive MC infiltration differed between breast cancers of respective molecular subtypes in both intratumoral area as well as at the invasive margin, and that higher MC numbers were associated with less aggressive cancer types. Similar to our results, della Rovere et al. observed high MC density in breast cancer expressing high levels of hormone receptors. As a result, the authors considered MC infiltration in this neoplasm as a protective factor against tumor progression, potentially due to MC cytolytic activity against malignant cells [19, 20]. Raica et al. noted that density of intratumoral, but not peritumoral, tryptase-positive MCs was higher in luminal A, luminal B, and HER2-positive breast cancers compared to basal-like breast cancers [21]. This was partially analogous to our results, which suggested that non-luminal HER2-positive subtype was associated with low tryptase-positive MC content. In the quoted study, significant correlations between peritumoral tryptase-positive MCs and lymphatic microvessel densities were found in luminal A and basal-like cancers. Such observation might indicate MC involvement in lymphangiogenesis and lymphovascular spreading of breast cancer, particularly of luminal A type [21]. Other studies also outlined the correlation between tryptase-positive MCs and microvessel density in breast cancer [22, 23].

We observed that all analyzed populations of MCs correlated positively with ER and PR expression and negatively with mitotic index. Additionally, tryptase-positive MCs both of the intratumoral area and at the invasion front were negatively associated with tumor size, while tryptase-positive as well as intratumoral chymase-positive MCs showed an inverse correlation with Ki67 expression. These findings supported the aforementioned hypothesis of the protective role assumed by MCs against cancer progression. Similarly, other studies also suggested a negative correlation of tryptase-positive MCs with tumor size [24], along with a positive correlation with PR [25] and ER expression [24]. Although several studies failed to show independent prognostic significance of MCs in breast cancer [24–26], and few works have even shown that peritumoral MC infiltration was associated with poor short-term survival [27], MCs were still proposed by some authors as an additive favorable prognostic factor [19, 25]. It was further postulated that even a single MC in tumor surrounding might have a beneficial impact on the prognosis [28]. Rajput et al. observed a positive but not significant correlation between MCs and HER2 expression [28]. However, our study suggested that chymase- and tryptase-positive MC densities at the tumor front were associated with tumors that did not indicate HER2 overexpression. Some other studies observed an inverse correlation between tryptase-positive MCs and Ki67 expression [24], while others did not [19]. Contrary to our results and the aforementioned literature, Ranieri et al. did not find any associations between MC number and tumor size, histological grade, ER/PR status, or HER2 overexpression in early breast cancer [23].

Our study indicated that low- and intermediate-grade breast cancers contained high numbers of MCs in both intratumoral location and at the invasive margin. In consistence with our results, some authors reported that tryptase-positive MCs correlated negatively with tumor histological Elston grade [24, 25]. A plausible explanation could be that low-grade breast cancer elicited more effective innate immune response, or that high-grade cancer suppressed such response. Strikingly, Xiang et al. observed more numerous peritumoral MCs in G3 breast cancers than lower grades, and reported more intensive tryptase immunostaining in the surrounding of node-positive tumors as compared to node-negative ones. In this experimental study, tryptase itself did not increase proliferative activity of breast cancer cell lines. However, in the presence of heparin, tryptase increased cancer cell migration and expression of activated MMP-1. As tryptase was activated by low pH and heparin, the authors concluded that tryptase promoted metastatic spread after microcirculation failed to remove acidic substances. This could potentially explain the higher MC count in more aggressive, more rapidly growing, grade 3 carcinomas observed in the study [29]. The findings from other immunohistochemical studies in breast cancer are summarized in Table 5.

**Table 5:** Immunohistochemical studies, which evaluated mast cells in breast cancer

Authors	Material	Mast cells’ marker	Conclusions	Reference
Bowers H. et al., 1979	Axillary lymph nodes of 43 breast cancer patients	Toluidine blue	Higher MC number is associated with better patients’ survival	[30]
Samoszuk M., Corwin M., 2003	35 breast cancer tissue sections of varying stages	Tryptase	A tendency toward peritumoral accumulation of MCs in preinvasive and intratumoral accumulation in invasive tumors	[31]
Amini RM. et al., 2007	234 invasive breast cancer tissues	Tryptase	MCs are associated with estrogen receptor positivity and low tumor grade	[25]
della Rovere F. et al., 2007	50 cases of invasive ductal breast cancer	Alcian blue	Higher MC content is associated with high hormone-receptive cancers	[19]
Ribatti D. et al., 2007	80 sentinel lymph nodes of breast cancer patients	Tryptase	Higher MC number in micrometastatic lymph nodes; MC quantity increases with angiogenesis	[32]
Rajput A. et al., 2008	4444 invasive breast cancer tissues	CD117	Presence of MCs in tumor stroma associated with better patients’ survival	[28]
Ranieri G. et al., 2009	88 breast cancer patients’ biopsy specimens	Tryptase	MCs are associated with angiogenesis	[23]
Xiang M. et al., 2010	80 breast cancer tissues	Tryptase	MC number positively correlated with tumor grade and was associated with nodal involvement	[29]
Löfdahl B. et al., 2012	190 lymph-node-negative breast cancer tissue samples	Tryptase	Negative associations between MC number and adverse prognostic factors	[24]
Raica M. et al., 2013	55 ductal invasive breast cancer tissues	Tryptase	Interplay between MCs and lymph vessels is specific for each molecular subtype of breast cancer	[21]
Marech I. et al., 2014	105 cases of breast cancer	Tryptase	Mast cell tryptase is involved in angiogenesis	[22]

Although the role of MCs in breast cancers has been investigated by several authors, the obtained results appeared to be ambiguous. Roy et al. [33] used an experimental model of arthritic mice for their study, which showed an elevated number of MCs within primary mammary tumors and at the sites of metastasis in comparison with the control group. This may be explained by the increased MC migration toward tumor and their activation within malignant lesion. MCs were suggested to attract stem cell factor (SCF) expressing breast cancer cells, thus facilitating the spread of the tumor. As SCF/c-kit signaling is considered to be one of the most potent chemoattractants and activators of MCs, SCF-positive neoplastic cells contributed, in turn, to subsequent infiltration, differentiation, and survival of MCs, which would eventually enhance metastatic potential of breast cancer [33]. Samoszuk et al. reported that MCs could counteract tumor hypoxia by releasing anticoagulants, which improved the blood flow. The authors also noted that tryptase-positive MCs in early breast cancer were more abundant in peritumoral stroma, while in invasive tumors, MCs were more extensively located within tumor tissue [31]. In the skin of breast cancer patients, chymase- and tryptase-positive MCs increased collagen production by interacting with dermal fibroblasts [34, 35]. It was also shown that MC tryptase has the capability to modify breast cancer microenvironment by converting fibroblasts into activated myofibroblasts, which, in turn, may promote tumor development. However, the accumulation of degranulated MC_T_ at the invasion margin was interpreted as an evidence for protective role against cancer growth [36]. Bowers et al. observed significantly higher MC number in axillary lymph nodes of breast cancer patients who survived for longer than 60 months post-mastectomy, in comparison with patients with a shorter survival time span. As a result, the authors postulated that MCs might be involved in host tumor resistance [30]. In contrast, higher MC and microvessel counts in sentinel lymph nodes with micrometastases as compared to non-metastatic sentinel lymph nodes could suggest the participation of MCs in metastasis formation [32].

Mast cells were also investigated in other types of cancer. The MC count in squamous cell carcinoma of the lip was found to be higher compared to that in normal tissue. The distribution of MCs in this neoplasm differed with reference to location: within the tumor nest, MC_T_ prevailed over MC_TC_ cells, while MC_TC_ predominated at the tumor front. It was postulated that the latter might influence cancer invasion [37]. Mast cells displayed different phenotypes in normal, hyperplastic, and malignant prostate tissues, thus suggesting alteration in MC phenotypes and their involvement in pathogenesis of prostate cancer. Moreover, peritumoral tryptase- and chymase-positive MCs correlated with increasing Gleason score [38]. In the cervix, the overall MC level was stable in pre-cancer, but increased significantly in invasive cancer. The prevailing phenotype of mast cells was MC_T_, and the authors hypothesized that this population may stimulate neovascularization and promote tumor progression and metastasis [39]. In addition, in patients on hemodialysis with renal cell carcinoma, MC_T_ were also reported to predominate; an elevated SCF expression in specimens from hemodialyzed patients could potentially account for this MC_T_ increase. MC_T_ density correlated positively with proliferative index and PAR-2 expression in tumor cells [40]. Melanomas were noted to display lower numbers of both chymase- and tryptase-positive intratumoral MCs as compared to common and dysplastic nevi. Interestingly, the number of these cells increased from common to dysplastic nevi. The authors suggested that the observed decrease of MCs in malignant melanoma might be due to the self-sufficiency of this neoplasm to induce neoangiogenesis or to break the host defense barrier [41].

Several authors focused on the associations between MCs and angiogenesis, a phenomenon linked to progression in various neoplasms. In non-small cell lung carcinoma, MC_TC_ correlated with blood vessel count both inside the tumor and at the invasive margin. In contrast, MC_T_ number correlated with blood vessel count only at the invasive margin, potentially due to angiogenesis being associated mainly with MC_TC_ density [42]. In an experimental mice skin cancer model, de Souza et al. observed that tumor MCs were recruited to the tumor microenvironment at their immature state, and that the number of both immature and mature MCs increased parallel to cancer progression. At early phases of tumor development, tryptase promoted neoangiogenesis, while in later stages it modulated vessel growth. Both chymase and tryptase expressions increased during tumor progression, and correlated with either MC maturation or new vessel formation, indicating the involvement of these two proteases in cancer progression [43]. Similarly in gastric carcinoma, tryptase- and chymase-positive MCs increased with stage and grade, and were associated with neoangiogenesis [44]. In colorectal adenocarcinoma, tryptase-positive MCs were found mainly in the immediate vicinity of blood vessels. However, as some of the tumor vessels lacked associated inflammatory cells, it was probable that inflammatory infiltration was not required for the induction of angiogenesis [45]. In contrast, neither tryptase- nor chymase-positive MC densities were related to microvessel counts in mesothelioma, though tryptase-positive MCs were associated with a better overall survival rate and a longer time till progression [46].

As it might be inferred from the aforementioned studies, MC contribution to tumor progression was observed in many neoplasms [12, 37, 38, 41, 43, 44]. However, in some tumors, MCs were regarded as protective factor [46], with their undefined role in breast cancer [24, 25, 29]. In various cancers MC distribution [37], as well as their prognostic significance, may vary depending on MC intratumoral [40, 41] or peritumoral [38] location, and in some cancers their increasing malignancy was reported to be associated with MC phenotype alteration [37, 38]. In breast cancer, an increase in the number of non-degranulated MCs from normal to malignant tissue was observed [36]. MC functions are strongly dependent on microenvironmental factors, and both cytokines as well as hormones may affect even mature MCs and influence their number, activation, suppression, mediators’ content, and phenotype [4, 47, 48]. Thus, it is not unlikely that on the basis on their functions and phenotype, in various cancers, the existence of several subpopulations of MCs could be considered, which might be partially analogous to distinction between M1 and M2 macrophages [47, 49].

In conclusion, our study outlined the associations of MCs with positive prognostic factors in breast cancer. We also observed that the breast cancer molecular subtypes differed in their chymase- and tryptase-positive MC content. However, further investigation is required to elucidate their impact on the breast cancer prognosis.
